# Acute tubulointerstitial nephritis/drug induced acute kidney injury; an experience from a single center in Pakistan

**DOI:** 10.15171/jrip.2016.04

**Published:** 2016-02-26

**Authors:** Rubina Naqvi, Muhammad Mubarak, Ejaz Ahmed, Fazal Akhtar, Anwar Naqvi, Adib Rizvi

**Affiliations:** ^1^Sindh Institute of Urology and Transplantation, Karachi, Pakistan

**Keywords:** Acute kidney injury, Tubulointerstitial nephritis, Aminoglycoside toxicity

## Abstract

**Introduction:** There is no information in literature specifically on the prevalence and clinicopathological characteristics of acute tubulointerstitial nephritis/drug induced acute kidney injury (AKI) from Pakistan.

**Objectives:** We aim to report a series of cases from patients developing AKI after exposure to some medications or finding of interstitial nephritis on histopathology.

**Patients and Methods:** This is an observational study of patients identified as having AKI after exposure to medications. AKI was defined according to RIFLE criteria and all patients fell from risk to loss category on arrival. On ultrasonography, all patients had normal size non-obstructed kidneys. Renal biopsy findings were consistent with tubule interstitial nephritis.

**Results:** Mean age of patients was 36.41 ± 17.40 years. Among total of 155, 80 were male and 75 female. Regarding drugs, most common was exposure to aminoglycoside in 34 (22%) followed by use of non-steroidal anti-inflammatory analgesics in 28, contrast induced agents in 11. Renal biopsy was performed in 58 patients. In half of these, insulting agent was not known and in rest either multiple medications were ingested or there was denial to substance use or recovery was delayed despite discontinuation of responsible medication. Renal replacement therapy was required on arrival in 119/155 (hemodialysis = 115, peritoneal dialysis = 4) cases. Complete renal recovery was observed in 71%, while 7.7% expired during acute phase, partial renal recovery was seen in 15% and 5% disappeared after first discharge from the hospital.

**Conclusion:** Tubulointerstitial nephritis may occur with many drugs of common use. Early and intensive efforts must be made to consider and then timely correct the injury to the kidney.

**Implication for health policy/practice/research/medical education:**

Tubulointerstitial nephritis is a common cause of acute kidney injury (AKI) throughout the world. Its true prevalence is unknown. The spectrum of drugs implicated varies from country to country. This study was carried out to determine the clinicopathological profile of patients with drug induced AKI or TIN at a single center in Pakistan. It included 155 patients over 25 years of study and highlighted the most commonly implicated drugs. Bringing forward this issue may guide the physicians to use such drugs with immense care and refer patients with renal involvement to renal care centers at early stage. Timely cessation of culprit medicine and addressing renal injury may prevent patients from progressing to end-stage renal disease (ESRD).

## Introduction

The kidney is the primary route of excretion for most of the drugs; many of these may cause damage to the kidney. There can be two mechanisms of kidney injury; the major mechanism is hypersensitivity to particular drug and the less common mechanism is direct drug toxicity. Nephrotoxicity may take several forms leading to renal vasoconstriction and reduced glomerular filtration rate (GFR) as a result. Defective renal tubular function is also a frequent event as proximal tubular cells are particularly susceptible to injury because of high capacity of transporting substances from urine to blood. Aminoglycosides have been reported as the leading cause of antibiotic induced nephrotoxicity few decades ago (1). Reports of nephrotoxicity from non-steroidal anti-inflammatory drugs (NSAIDs) are common; they remain one of the most common causes of drug induced acute kidney injury (AKI) especially when used in different combinations (2). Rifampicin, a commonly used anti tuberculous drug, has also been reported to cause tubulointerstitial nephritis (TIN) and AKI (3,4). Contrast agents used for many radiological examinations have also been reported to cause TIN (5). Reports on TIN and AKI with penicillin, methicillin (6), cloxacillin (7), and methoteraxate (8) are found in literature over last many decades. Herbal medications that are unidentifiable on most occasions are known to cause TIN (9).

## Objectives

We aim to report a series of cases from patients developing AKI after exposure to some medications or finding of interstitial nephritis on histopathology.

## Patients and Methods

This study is based on a cohort of 155 patients with AKI after exposure to different medications from a retrospective chart review of all patients admitted to the Sindh Institute of Urology and Transplantation (SIUT), Karachi, Pakistan between January 1990 and December 2014. AKI was identified and staged according to RIFLE criteria (10). Patients with preexisting kidney disease and diabetes were excluded.

Diagnosis of drug exposure was based on history of getting these drugs before developing AKI. Majority of patients were given these drugs as treatment tool for some ailment while some took as self-medication (especially NSAIDs and herbal medications).

TIN was suspected on the basis of the history, and in some patients presenting features which included fever and rash.

On the day of admission to this hospital following laboratory tests were carried out in all patients; complete blood count (CBC) with differential white cell count, blood urea, serum creatinine, serum electrolytes, liver function tests, blood sugar levels, and urinalysis. Urine for eosinophils, serum lactate dehydrogenase, creatine phosphokinase, and uric acid were done in selected cases. Eosinophils in urine were checked by staining slide with Leishman stain and looking under the light microscope.

Ultrasonography was performed on the day of hospital admission in all patients, and all had normal size non-obstructed kidneys.

Renal biopsy was performed in 58 cases, and evaluated with light microscopy (LM). Routinely,10 serial sections were cut and stained by hematoxylin and eosin (H&E), Masson’s trichrome stain, periodic acid-Schiff (PAS), and silver stain (Gomori’s methenamine silver, GMS), as described in detail in our previous reports (11).

All patients were followed up till death, complete renal recovery or development of chronic kidney disease (CKD), except for those who were lost to follow-up.

### Ethical issues

1) The research followed the tenets of the Declaration of Helsinki; 2) informed consent was obtained; and 3) This study was approved by the Ethics Committee of Sindh Institute of Urology and Transplantation.

### Statistical methods

Statistical analysis was done on SPSS version 20.0 (SPSS Inc., Chicago, IL, USA). Descriptive statistics of mean ±standard deviation (SD) were used for continuous variables and numbers (percentages) for categorical variables. *P*<0.05 was taken as the significant level.

## Results

A total of 155 cases with AKI secondary to drug hypersensitivity or toxicity were registered during the study period. There were 80 (51.6%) males and 75 (48.4%) females with mean age of 36.41 ± 17.40 years. Exposure to aminoglycoside was most common finding observed in 34 (21.93%) patients, followed by use of NSAIDs in 28 (18%). Frequency of different drugs used is given in Table 1. Fever was found in 46.45% patients and rash was observed in 21.93%. Average time to insult was 14±10 days on arrival. Laboratory values on the day of reporting at this hospital are given in Table 2. Urinalysis was available in 131 patients; dipstick revealed 1-3+ protein in 86 patients, while 8 had 4+ protein. Microscopy revealed hematuria in 109 cases. Eosinophils in urine were checked in 43 patients and found positive in 8 patients.

**Table 1:** Pharmaceutical agents causing tubulointerstitial nephritis and AKI

**Medication used**	**No. of patients**	**Percent**
Aminoglycoside	34	21.93
NSAIDs	28	18.06
Contrast agents	11	7.09
ATT	8	5.16
Multiple drugs	6	3.87
Penicillin	4	2.58
Chemotherapeutic agents	3	1.93
Others (herbal, fansidar)	3	1.93

Abbreviations: AKI, acute kidney injury; NSAIDs, non-steroidal anti-inflammatory drugs; ATT, anti-tuberculosis therapy (rifampin, isoniazid, pyrazinamide and ethambutol).

**Table 2:** Clinical and laboratory parameters of the study population

**Parameters**	**Values**
Days of insult (mean±SD)	14 ± 10.76
Fever (%)	46.45
Rash (%)	21.93
Hemoglobin g/dL (mean ± SD)	9.91 ± 2.79
TLC (mean ± SD)	14.50 ± 10.07
Eosinophilia (%)	28
Blood urea mg/dL	236.03 ± 100.08
Serum creatinine mg/dL	10.23 ± 5.28
Serum sodium mg/L (mean ± SD)	130.66 ± 8.08
Serum potassium mEq/L (mean ± SD)	4.48 ± 1.31
Dipstick protein 1-3+ (%)	59.35
Microscopic hematuria (%)	70.32
Eosinophiluria (checked in 43 patients)	Negative in 35

**Figure 1:**
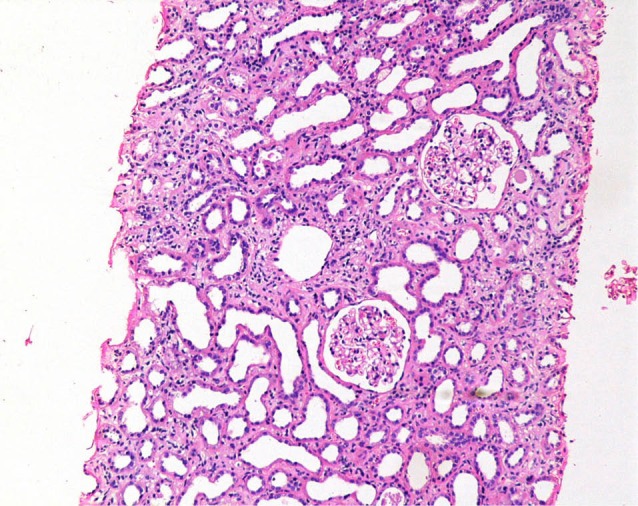
Low-power photomicrograph showing relatively normal glomeruli, severe acute tubular injury associated with interstitial edema and mononuclear cell infiltration in the interstitial are in a case of tubulointerstitial nephritis (Hematoxylin & Eosin ×100).

Renal biopsy was performed in 58 (37.4%) patients; in half of these, insulting agent was not known and in rest either there were multiple medications ingested or there was denial to substances use or recovery was delayed despite discontinuation of responsible medication. In patients, with diagnosis of acute TIN, glomeruli were found to be normal (Figure 1).

Renal replacement therapy was required in 76.77% patients. Complete renal recovery occurred in 71.61%, while 7.7% died during acute phase of illness; 14.83% achieved partial recovery and the rest 5.1% were lost for follow-up after discharge from the hospital. A total of 12 (7.7%) patients died during acute phase of illness; 6 had element of sepsis, 2 required mechanical ventilation (one with herbal medications had polyneuropathy, TIN was biopsy proven in this case), one died with hematemesis, one with bacterial endocarditis. Congenital heart disease was cause of death in one and 2 patients had severe bone marrow suppression. Both gave history of fansidar (sulphadoxine/pyrimethamine) as the responsible drug. Of these, 12 patients, 6 were exposed to aminoglycoside, 2 to fansidar, 2 to NSAIDs, one to penicillin and one to herbal drugs. Mean age of patients who died was 35±19.57 years (range: 14-70 years); eight were males, and four females.

## Discussion

Acute TIN broadly describes diseases of kidney which spare the glomeruli and affect primarily the tubulointerstitial compartment. Injury to renal tubular cells leads to expression of new antigens, infiltration of inflammatory cells and activation of different sets of cytokines, pro-inflammatory and chemo-attractant, both. The tubular damage results in tubular dysfunction and AKI may or may not occur. Regardless of the extent of damage, functional loss is usually reversible (12). Hypothetically any drug can cause hypersensitivity reaction and damage to the kidney but most commonly reported are aminoglycosides, penicillin, NSAIDs, rifampicin, contrast dyes, chemotherapeutic agents and herbal medications (1-9). A decline in aminoglycoside toxicity has occurred with the development of less nephrotoxic antimicrobial agents. In current study, we found 25 out of a total of 34 aminoglycoside toxicity patients during the initial 10 year period and remaining 9 cases spread over the later 15 years.

Some of our patients had history of exposure to multiple antimicrobials along with analgesics. It is a well known fact that combination of therapies especially if NSAIDs are combined with angiotensin converting enzyme inhibitors or receptor blockers or with diuretics, the chances of AKI increase manifold (2).

Most patients with hypersensitivity reaction and allergic TIN recover normal renal function after cessation of offending medication. In the present study, we performed renal biopsy in selected cases, where either they exhibited delayed recovery after discontinuation of drug, or the patients denied of the use of any drugs before reaching to this institution or exact description of used drug was not available.

TIN may progress to end-stage renal failure (ESRF) and lifelong renal replacement therapy may be needed (12). In our studied population, approximately 15% patients’ revealed partial renal recovery and another 5% were lost to long-term follow-up.

Use of traditional herbal medications is not uncommon in our society, but majority of them do not report to modern medicine facilities or if by any means reach there, they usually do not reveal their practice. One of our studied patients, who died while on mechanical ventilatory support, had consumed some herbal remedies and renal histopathology in this particular patient confirmed the diagnosis of TIN.

Mortality directly related to TIN is rare. Six patients in our series died of sepsis which is a frequent angio-access related problem in our setup. One of our patient had massive gastrointestinal bleed and could not survive it, while two had marked marrow suppression after taking “fansidar” (sulphadoxine/pyrimethamine), and renal histopathology revealed TIN in these cases.

## Conclusion

In conclusion, TIN may occur with many drugs of common use. Early and intensive efforts must be made to consider and then timely correct the injury to the kidney.

## Limitations of study

Many of our patients where history is suggestive of drug induced injury unable to tell the name of drug or show the prescription thus cannot be addressed.

## Authors’ contribution

RN: Data collection, design, literature search, statistical analysis, manuscript writing. MM: Helped in histopathological evaluation, description of methods and manuscript writing. EA: Helped with patient management and decisions towards management. FA: Helped with patient management and decisions towards management. AN: Deputy Director of Institute, helped in provision of funds towards all steps of patient management. AR: Director of institute, helped in provision of funds towards all steps of patient management.

## Conflicts of interest

There were no points of conflicts.

## Ethical considerations

Ethical issues (including plagiarism, data fabrication, double publication) have been completely observed by the authors.

## Funding/Support

None.
